# Review of the Article Entitled “Infants and Their Food”

**Published:** 1874-05

**Authors:** G. Newkirk

**Affiliations:** Low Point, Ill.


ARTICLE III.—*Review of the Article entitled "Infants and their Food."* By G. NEWKIRK, M.D., Low Point, Ill.

The paper in question published in the March No. of the CHICAGO MEDICAL JOURNAL appears to me to call for a few comments by way of criticism. It commences with the report of a case, which is at once interesting and instructive, the description thereof being well written. The previous treatment of the case was properly criticised, and the course of diet and regime directed by the author, eminently proper under the circumstances as described.

We can readily understand how with natural food and sleep "came the angel of health and brought joy to the parents, life to the sufferer, and to him a great lesson," but we may fail entirely to see the legitimate fruits of such lesson in that which follows from the author's pen.

Let us see. A child is deprived of all proper food and as a matter of course it starves! The physician finds it in a starving condition, being *dosed* but not *fed!* Milk—"cow's milk from the pan"—without any addition to or subtraction from it, fresh from nature's laboratory, is given, threatened failure of the powers of life is averted, and the functions of growth renewed. That the preceding attendant had failed to discover the simple condition of starvation, and prescribe the still more simple remedy of food, was certainly to his discredit. That our author did what his predecessor had failed to do, speaks well for his practical sense.

But we cannot from the author's observation of this case infer that the addition of sufficient water to the milk used for the purpose of equalizing the relative proportions of fluid and solid, in accordance with the constitution of human milk, or that the addition of two per cent. of sugar would have been otherwise than beneficial; neither that the addition thereunto of sweet almond or cod-liver oil—both so liable to detestable adulteration—as recommended by the author further on, would have not proved injurious. We are decidedly of the opinion that *no addition* would be far preferable to that which he recommends but does not tell us he has tested practically.

Every member of the profession must have seen results equally tangible with those here reported where the usual additions were made. When we come to consider quantitative analysis we can only approximate a standard with regard to organic fluids. Circumstances of a varying nature, such as diet, exercise, emotion and constitutional tendencies, must always vary the constitution of the secretions, including milk. No two chemists can give us the same tables, and for obvious reasons.

Liebig gives us a general and simple analysis, classing together the sugar and the oils as follows:

| | Nutritive. | Respiratory. |
|---|---|---|
| Cow's milk contains, . . . . . . . . . . . . . . | 10 | 30 |
| Human milk contains, . . . . . . . . . . . . . | 10 | 40 |

(*Attfield.*)

| | Water. | Solids. | Casein. | Sugar. | Butter. | Salts. |
|---|---|---|---|---|---|---|
| Human, . . . . . . | 889 | 111 | 40 | 44 | 27 | 2 |
| Cow, . . . . . . . . | 864 | 136 | 55 | 38 | 36 | 7 |

(*Pereira & Lehmann.*)

| | Water. | Casein. | Butter. | Sugar. | Salts. |
|---|---|---|---|---|---|
| Cow's milk, . . . . . . . | 870.2 | 44.8 | 31.3 | 47.7 | .6 |

(*Simon.*)

| | Casein. | Butter. | Sugar. | Salts. |
|---|---|---|---|---|
| Human milk, . . . . . . . . | 35 | 25 | 48 | 2 |

Observe what wide differences! According to Pereira & Lehmann the proportion of casein in cow's milk is represented by 44.8, according to Simon, 68; a difference of 23.2! By balancing the different tables as nearly as we may, we arrive at the following conclusions: 1st, that the excess of casein in cow's milk as compared with that of woman is about twenty per cent., or about eight parts in 1,000; 2nd, that sugar is *deficient* in nearly the same ratio; 3rd, that the amount of fat is about equal—according to Attfield we see that the balance stands very much in favor of the cow's milk. According to Simon (whose table our author quotes) the amount of butter in human milk is but 25 less than any authority I can find gives for cow's milk.

The amount of butter contained in milk varies greatly in different animals of the same species, as all butter makers know. The butter making qualities of the cow do not depend so much on the *quantity* as the *quality* of her milk. The *quality* is, in turn, dependent on two conditions, viz., *amount* of cream or butter, and the proportion of its oils. The last of the milking is richer in butter than that first drawn.

The ratio of the solid to the fluid increases with the age and requirements of the animal to be nourished; therefore, other things being equal, the milk of the farrow cow is richest in tissue-making substances, more especially of casein.

In the healthy human being corresponding conditions must naturally obtain. A gradual change must ensue in the wants of the growing child, and the constitution of its food should in the nature of things bear a gradual modification. Nature here takes the initiatory by gradually increasing the *quality* of milk to meet the requirements of increasing strength, while *quantity* is diminished to admit the digestion of other and more solid aliment. Bear in mind, however, these rules hold good in the healthy mother only, who has not been reduced in strength by multitudinous cares and labors, or an impoverished diet superadded to a prolonged lactation.

Again, we notice that all authorities agree, with but little variance, as to the disproportion existing in the amount of sugar in the two kinds of milk. This disproportion is probably less varying than that of any other element contained in milk; the tables at my command varying from 31 to 38 in that of the cow, and from 44 to 48 in that of woman, showing a difference of about twenty-five per cent.

The young infant relies upon milk for both food and drink; hence it is important that the proportions of fluid and solid should correspond with its wants. We add a little water to make good these proportions, and not, as our author observes, for the purpose of "diluting casein." However, it is not true, as he states, that casein in milk is not dilutable with water; alone it is not, but in milk it is in a compound with soda, hence as divisible as any salt already in solution. (Youman's Chemistry, p. 390.) As to the exact quantity to be added, experience as well as sound philosophy justifies us in being governed by the age and general health of the child, as well as its digestive tone. As a rule, the strength of food upon which the child will grow, and bears without vomiting or diarrhœa, is for the time being proper for it. With increasing age, quality as well as quantity should be increased. We do not add sugar, as our author states, to promote the digestion and assimilation of casein, but simply and alone to supply a want, and in such proportion as nature demands. We presume that whatever nature furnishes in the mother's milk, she has use for in the system of the child, both in amount and kind. Just how sugar is assimilated we cannot tell, but this we know, that it is *essential;* a *carbo-hydrogen*, hence, respiratory, and a brain and nerve food; that as an aliment it can largely take the place of fat.

Inasmuch as the young and nursing infant eats no starch—out of which sugar is made—nature provides the sugar of milk, and if we accept and follow out her teachings we are bound to supply at least an equal amount to the hand-fed child. In accordance with what seems to be the physiological indications here set forth, we find the natural appetite craving sugar. If a natural and unperverted appetite is in any instance to be relied on as an indication of systemic wants, certainly it may be here.

We have yet to learn that simple redness of the mucous surface, which always occurs during digestion, is an inflammatory indication, as stated by the writer. The author should certainly be able to draw a distinction between inflammation and a physiological afflux of blood, ever taking place in the part temporarily called into increased activity.

We heartily concur with him in his denunciation of elixirs and all unknown and advertised compounds; have no objection to the theory of Lehmann, "that a certain, though small quantity of fat is indispensable to the metamorphosis and solution of nitrogenous articles of food during the progress of gastric digestion;" adding, however, that there can be no doubt but the "small quantity" can be found in fresh cow's milk at any time, and without the addition of sweet almond or cod-liver oil.

Considering that the study of carcinoma and tuberculosis cannot properly be entered into in a paper of this kind, and that the random shots taken by the author at these and various other questions are far from the mark of the high calling in which he thinks himself engaged, we will pass them by and proceed to notice his experiments with bottles and milk. We will repeat his statement of them briefly:

No. 1. Cow's Milk, with vinegar.
No. 2. " " " almond oil and vinegar.
No. 3. " " " water (half and half) and vinegar.

He then proceeds to tell us that "in No. 1 the casein separated slowly from the whey *and settled on the bottom of the vial*," (mark the emphasis), "the precipitation being not quite complete."

"In No. 2 the casein separated completely, but the *coagulum was decidedly more flocky, and, what is very remarkable, it floated on the clear whey.*" (Mark the emphasis.)

In No. 3, according to his observation, precipitation failed to take place.

*Remarks.* The rapidity and completeness with which coagulation of casein takes place under these circumstances depends upon the amount of acid added; in this case it was insufficient, "precipitation being not quite complete." In the repetition of his experiment by himself he states that there was a more distinct separation, owing to the increased amount of vinegar added. Casein thus precipitated has scarcely specific gravity enough to carry it downward; again, it holds a large portion of the butter entangled in its meshes, which tends to float it; however, if left undisturbed, it finally settles to the bottom of the vial.

In No. 2 we deny that coagulation could have been any more complete. Let any one make the test and see for himself the fallacy of the author's deductions, so called. We can easily account for the fact which the author considers so very remarkable, viz.: that "*the coagula floated on the clear whey.*" The oil of almonds, in addition to the butter, becoming admixed or entangled with the flocky precipitate, floated it to the top, as we will make plain a little further on. There were not two distinct precipitates, one which floated, and the other which fell; finer flakes or more regular in outline, or which were first at the bottom when coagulation commenced, failed to entangle sufficient oil to float them, and, as in No. 1, slowly fell.

In No. 3 we are utterly at a loss to understand how the gentleman could so fail in correct observation.

In order to thoroughly test the correctness of the author's theories, as well as his facts—so called—let any one who will, institute the following experiments: Add to an ounce of fresh milk twenty drops of almond oil and sufficient vinegar to coagulate the casein. If the mixture is shaken while coagulation is going on, or afterward, the oil will float the curd, or the principal part of it. If the mixture stands for a few moments previous to precipitation, the oil will quickly find the surface and the coagula will slowly settle. The same amount of vinegar added to the milk without the oil will just as readily throw down the curd, and there will be no difference in the appearances of either curd or whey as compared with the first experiment. We took skimmed milk, from which nearly all the butter had been taken; to three drachms of this we added four drachms of water and two scruples of vinegar, and coagulation at once took place, leaving the transparent water and whey. We repeated the experiment with *one drachm of skimmed milk to six drachms of water*, and this, with a few drops of vinegar, precipitated more rapidly than the first! There was no difference in the appearance of the precipitate; it settled more readily, not because it was heavier, but because there was very slight, if any, admixture of *oil*. In either instance, on the addition of a small amount of oil after coagulation and the mixture being shaken, the oil would carry the curd upward and retain it there. According to the author's idea the precipitate resulting from such a quantity of menstruum would have been too heavy to float.

Thus we find that our friend's experiments prove nothing. With all due courtesy we must beg leave to remark that his facts—so called—are as false as his theories. The article in question only goes to show how far a little knowledge—diffused surface reading—with a few coincidences and a disposition "to revolutionize," may carry a sensible man toward the climax of absurdity even unto the claimed demonstration of absolute nonsense.

To conclude, we maintain our adherence to the following rules:

1st. That casein in milk is dilutable as well as other solid constituents, and that ordinary dilution does not interfere with digestion; therefore, that it is proper to dilute cow's milk with water in proportions varying according to the age, health, and digestive tone of the child to whom it is to be fed.

2nd. That the addition of sugar to make good the percentage as in human milk, is proper, physiological, and necessary; that it does not tend to intestinal irritation nor interfere with digestion, even though given in slight excess of the actual requirements of the organism, being easily appropriated and of an unirritating nature.

3rd. That no addition of any extra fat is necessary to cow's milk when properly selected; that if any should be given it should be in the form naturally belonging to milk, viz., cream; that the milk last drawn from the cow contains as much and usually more fat than human milk.

4th. That, while the system of the infant may reject and dispose of as residue any supply of proximate principles moderately in excess of its wants, it *cannot supply an absolute deficiency*. Supply should at least be equal to demand.

Finally, we wish to express our admiration of the sentiment quoted—"The hand that rocks the cradle is the hand that rocks the world"—whether it be that of noble woman, the boy who has nothing else to do, or the baby who is old enough to rock itself. We think the sentiment more applicable, however, to Him who rocks the cradle of the deep.

